# Identification of a Novel Recombinant Type 2 Porcine Reproductive and Respiratory Syndrome Virus in China

**DOI:** 10.3390/v10040151

**Published:** 2018-03-27

**Authors:** Long Zhou, Runmin Kang, Bo Xie, Yiming Tian, Xuan Wu, Xuebin Lv, Xin Yang, Hongning Wang

**Affiliations:** 1 School of Life Science, Sichuan University, Animal Disease Prevention and Food Safety Key Laboratory of Sichuan Province, Key Laboratory of Bio-Resources and Eco-Environment, Ministry of Education, 29# Wangjiang Road, Chengdu 610064, China; 2014322040041@stu.scu.edu.cn (L.Z.); 2015322040022@stu.scu.edu.cn (Y.T.); 2016322040030@stu.scu.edu.cn (X.W.); xinyang@scu.edu.cn (X.Y.); 2 Sichuan Animal Science Academy, Sichuan Provincial Key laboratory of Animal Breeding and Genetics, Chengdu 610066, China; angelina_0708@hotmail.com (R.K.); lvxuebin@21cn.com (X.L.); 3 Chengdu Chia Tai Agro-Industry & Food Co., Ltd., Animal Healthy Disease Service, Gongping Town, Wenjiang District, Chengdu 610081, China; bobosky123@163.com

**Keywords:** porcine reproductive and respiratory syndrome virus, sub-genotype, phylogenetic analysis, recombination

## Abstract

Since the emergence of NADC30-like porcine reproductive and respiratory syndrome virus (PRRSV) in China in 2013, PRRSVs have undergone rapid evolution. In this study, a novel variant of PRRSV strain (designated SCcd17) was successfully isolated from piglets with clinical signs in Sichuan Province in China in 2017, and the complete genomic sequence was determined. The genome of this new isolate was 15,015 nucleotides (nt) long, and comparative analysis revealed that SCcd17 exhibited 90.2%, 85.2%, 84.9%, and 84.0% nucleotide similarity to PRRSVs NADC30, JXA1, CH-1a, and VR-2332, respectively. Phylogenetic analysis indicated that the SCcd17 strain was classified into the NADC30-like sub-genotype, in which all the strains contained the unique discontinuous 131-amino acid deletion in nonstructural protein 2 (nsp2) when compared to VR-2332-like viruses. Notably, extensive amino acid substitutions were observed in nsp2 and a unique single amino acid deletion at position 33 of the GP5 is being described for the first time. Strikingly, recombination analysis revealed that SCcd17 was the result of recombination between the NADC30-like, JXA1-like, and VR-2332-like strains at five recombination breakpoints: nsp1α (nt 641), nsp3 (nt 5141), nsp10 (nt 9521), open reading frame 3 (ORF3) (nt 12,581), and ORF4 (nt 13,021). The genomic data of SCcd17 will be helpful for understanding the role of genomic recombination in the evolution of PRRSV.

## 1. Introduction

Porcine reproductive and respiratory syndrome (PRRS) has become an economically important disease in the global swine industry since its first identification in North America in 1987 [1]. The causative agent of this disease, the PRRS virus (PRRSV), is a single-stranded positive-sense RNA virus and is classified in the order *Nidovirales*, family *Arteriviridae*, and genus *Porartevirus* [2,3,4]. The genome of PRRSV is approximately 15 kb in length and encodes at least 12 open reading frames including ORF1a, 1b, 2a, 2b, and 3–7 [5,6,7]. The first two ORFs encode 16 nonstructural proteins (nsps) that are necessary for RNA replication: nsp1α, nsp1β, nsp2, nsp2TF, nsp2N, nsp3–6, nsp7α, nsp7β, and nsp8–12. ORFs 2–7 encode several structural proteins: the minor envelope proteins (GP2a, E, GP3, GP4, and GP5a), the major envelope protein (GP5), the unglycosylated membrane protein (M), and the nucleocapsid protein (N) [5,8,9].

PRRSV is classified into two genotypes, type 1 PRRSV (prototype strain Lelystad virus) and type 2 PRRSV (prototype strain VR-2332), which exhibit a divergence of approximately 60% at the nucleotide level [10,11,12]. In China, genotype 2 PRRSV has been circulating and has been predominant in the field since its initial emergence in late 1995 [13]. In 2006, a highly pathogenic PRRSV (HP-PRRSV) (prototype strain JXA1) caused outbreaks, which resulted in the death of more than two million pigs [14]. Despite the application of modified-live virus (MLV) vaccines for HP-PRRSV, the diversity of type 2 PRRSV has increased rapidly, with continued reporting of virulent PRRSV variants [15,16,17,18]. During 2013–2014, novel NADC30-like PRRSV strains that exhibit high nucleotide similarity with NADC30 (a moderately virulent strain reported in USA in 2008) have been identified in several provinces in China, adding to the complex situation and the genetic diversity of PRRSV in China [19,20,21].

PRRSVs in China have demonstrated a high rate of genetic change that has contributed to the appearance of diverse subpopulations that continually emerge to form new variants. To date, an overwhelming majority of the type 2 PRRSV strains in China can be divided into four sub-genotypes: JXA1-like, CH-1a-like, NADC30-like, and VR-2332-like sub-genotypes [22,23,24,25]. However, recombination events can occur among field strains or between field and MLV vaccine strains, further increasing viral diversity. For example, recently, recombination events involving NADC30-like PRRSV strains and JXA1-like strains (HENAN-HEB, JL580, HNhx, TJnh1501, and FJ1402) or VR2332-like strains (HENAN-XINX, Chsx1401, and HNyc15) occurred [19,26,27,28,29,30].

In the present study, we isolated PRRSV strain SCcd17 from piglets with clinical symptoms from a pig farm in Sichuan Province, China, in 2017. We performed genomic sequence analysis that revealed evidence of multiple recombination events among NADC30-like, JXA1-like, and VR-2332-like strains, which are currently circulating in China.

## 2. Materials and Methods

### 2.1. Sample Collection and Virus Isolation

The SCcd17 strain was isolated using porcine pulmonary alveolar macrophages (PAMs) from the lung tissues of diseased pigs collected from one pig farm in Sichuan Province, Southwestern China, in 2017. The pigs showed high fever and obvious respiratory signs. Lung samples were inoculated into PAMs and cultured in RPMI-1640 medium (Transgen, Beijing, China), supplemented with 5% fetal bovine serum (HyClone, South Logan, UT, USA) at 37 °C under a humid 5% CO_2_ atmosphere. The presence of the virus was confirmed by daily observation of cytopathic effects (CPE) and an indirect immunofluorescent assay (IFA) using anti-N protein of PRRSV monoclonal antibody, GTX (GeneTex, Irvine, CA, USA). The virus was purified twice by plaque assay and the PRRSV isolate SCwhn09CD (isolated in 2009 in Sichuan Province) was used as a positive control in the plaque assay [31]. The purified viruses were used for complete genome sequencing. The genomic sequences of PRRSV strain SCcd17 have been submitted to GenBank database and were assigned accession no. MG914067.

The collection of PAMs and virus-containing tissue samples in this study was performed in strict accordance with the guidelines for the care and use of laboratory animals approved by the Animal Ethics Committee (AEC) of Sichuan University (Chengdu, China, 15 January 2017, SYXK [Chuan] 2013-185).

### 2.2. RT-PCR Amplification and Genome Sequencing

For complete genomic sequencing, viral RNA was extracted from 200 µL of viral stocks using TRIzol reagent (TaKaRa, Dalian, China) according to the manufacturer’s instructions. First-strand cDNA was synthesized using random hexamers (TaKaRa, Dalian, China) following the manufacturer’s instructions. The complete genomic sequences of SCcd17 were amplified with fourteen overlapping fragments by reverse transcription-PCR (RT-PCR) as described previously [32]. The PCR products were purified and cloned into pMD19-T vector (TaKaRa, Dalian, China) following the manufacturer’s instructions, and then sequenced at least three times by the commercial service (Sangon, Shanghai, China) using Sanger sequencing approach. The 5′ and 3′ ends of the viral genome were amplified using a 5′/3′ RACE kit (TaKaRa, Dalian, China) according to the manufacturer’s instructions. The sequences of fourteen overlapping fragments from SCcd17 were assembled into full-length genome sequences using the SeqMan program in DNAstar 7.0 software (DNASTAR, Madison, WI, USA).

### 2.3. Sequence Comparison and Phylogenetic Analysis

Multiple sequence comparisons at the nucleotide and amino acid levels were performed using the MegAlign program in DNAstar 7.0 software and BioEdit program (v7.0.5, Borland, Scotts Valley, CA, USA). Evolutionary analyses were conducted in MEGA6 software (v6.06, Tempe, AZ, USA) and phylogenetic trees were constructed using the neighbor-joining method and the Maximum composite likelihood method. The bootstrap values were evaluated from 1000 replicates. The selected PRRSV reference sequence strains and their detailed information are shown in Table 1.

### 2.4. Putative Recombination Analysis

To identify potential recombination events, the complete genomic sequence of SCcd17 was compared with the sequences of JXA1 (HP-PRRSV), CH-1a (Chinese C-PRRSV), VR-2332 (North American prototype), and NADC30. The aligned nucleotide sequences of the complete genomes were analyzed using the SimPlot software (v3.5.1, JHK University, Baltimore, MD, USA) with a 200-bp window and a 20-bp step. In addition, a Recombination Detection Program v4 (RDP4) was used to confirm the putative recombination events and precise recombination breakpoints. Furthermore, phylogenetic trees based on each recombinant fragment were constructed to avoid phylogenetic biases derived from ignoring recombination events.

## 3. Results

### 3.1. Genome Characterization and Homology Analysis

The Strain SCcd17 was successfully isolated from the clinical samples and were subjected to whole-genome sequencing. The complete genomic sequence of SCcd17 is 15,015 nucleotides (nt) in length, excluding the poly (A) tail at the 3′ end. Genetic analyses revealed that SCcd17 exhibits 85.2%, 84.9%, 84.0%, and 90.2% nucleotide identity with PRRSV strains JXA1, CH-1a, VR-2332, and NADC30, respectively, and only 43.4% nucleotide identity with the European prototype Lelystad virus (LV, prototype of genotype 1 PRRSV), indicating that the SCcd17 strain belongs to type 2 PRRSV (Table 2).

In order to evaluate the genomic characteristics of the novel PRRSV isolates, the genome of SCcd17 was compared with that of the JXA1-like PRRSVs (JXA1, JXwn06, and TJ), VR-2332-like PRRSVs (VR-2332 and RespPRRSV MLV), CH-1a-like PRRSV (CH-1a), and NADC30-like PRRSVs (NADC30, JL580, CHsx1401, HENAN-HEB, and HNjz15) (Table 2). The 5′-UTR of SCcd17 is 189 nt in size, with 94.9–97.8%, 92.7%, 96.7%, and 90.3–92.2% nucleotide identity with JXA1-like, VR-2332-like, CH-1a-like, and NADC30-like PRRSVs, respectively (Table 2). The 3′-UTR of SCcd17 is 151-nt long, excluding the poly(A) tail, sharing 96.6–97.3% nucleotide identity with the NADC30-like PRRSVs, and 82.3–89.2%, 93.0%, and 88.5% nucleotide identity with JXA1-like, VR-2332-like, and CH-1a-like PRRSVs (Table 2), respectively, indicating a higher level of genetic variation within the 3′-UTR than the 5′-UTR. ORF1a and ORF1b are 7119 nt and 4383 nt in length, respectively, and together encode 16 nsps. Among nsps, nsp2 and nsp3 are the most variable protein products, with 23.6–92.3% and 32.8–90.4% nucleotide identity with the reference strain, respectively (Table 2). ORFs 2a to 7 cover about one-fourth of the genomic sequences, and encode the PRRSV structural proteins. GP2a, GP3, and GP5 encode the most variable protein products among the structural proteins, with 52.4–92.8%, 46.2–96.4%, and 52.7–88.6% nucleotide identity with the reference strains, respectively (Table 2).

### 3.2. Phylogenetic Analysis

**Figure 1:**
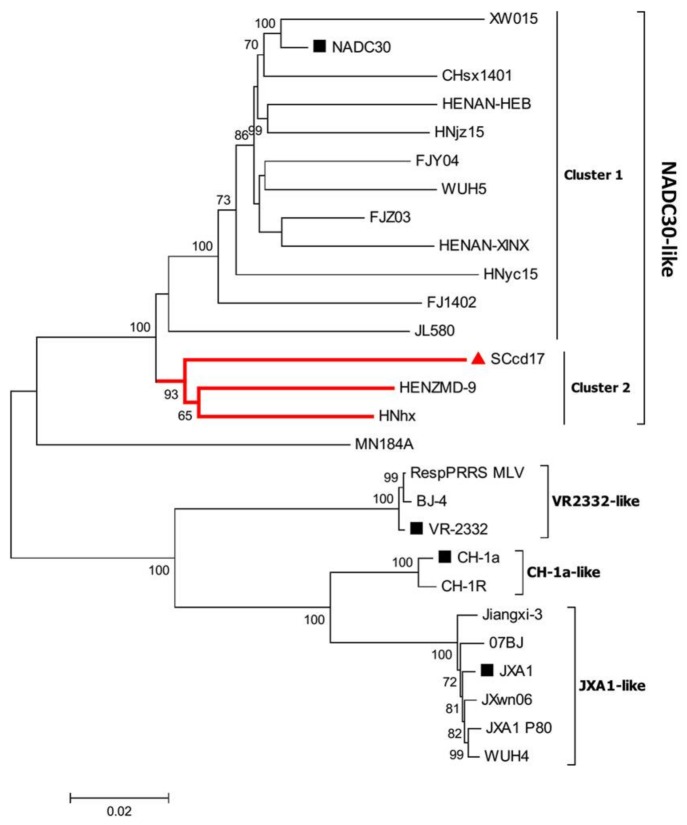
Phylogenetic tree based on full-length genomic sequence of SCcd17 isolate and PRRSV reference strains available in GenBank. The strain SCcd17 isolated in this study is labeled with “red triangle”. The representative strains are labeled with “black squares”. The numbers along branches are bootstrap values. The scale bar indicates the number of nucleotide substitutions per site.

Phylogenetic analysis of SCcd17 was performed based on the complete nucleotide sequences of 27 reference PRRSVs of type 2 available in GenBank (Table 1). The results showed that all of the genotype 2 PRRSV strains can be divided into four sub-genotypes: JXA1-like, VR-2332-like, CH-1a-like, and NADC30-like sub-genotypes (Figure 1). All other NADC30-like PRRSVs that have been previously reported in China were divided into two different clusters (Clusters 1 and 2). The SCcd17 isolate separated into Cluster 2, together with two other strains (HNhx and HENZMD-9), indicating that SCcd17 belongs to the NADC30-like sub-genotype.

Moreover, SCcd17, HENZMD-9, and HNhx strains showed a more distant phylogenetic relationship with the NADC30 strain than the other NADC30-like PRRSV isolates within Cluster 1 (Figure 1).

### 3.3. Amino Acid Analysis of Nsp2 and GP5

**Figure 2:**
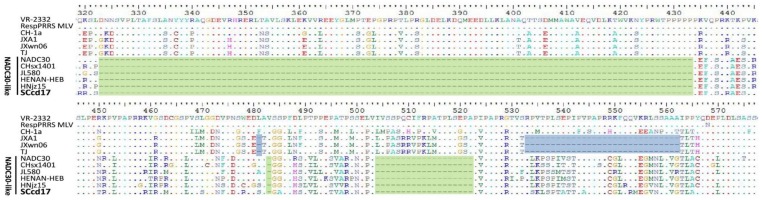
Analysis and comparisons of the nsp2 deduced from amino acid sequences. Deletions in SCcd17 and related NADC30-like strains are shaded green, and deletions in the Chinese HP-PRRSV strains are shaded blue. The conserved residues were hidden.

Nsp2 is the most variable non-structural protein of PRRSV, and comparison of the different sequences reveals that nsp2 contains different patterns of insertions and deletions. Amino acid alignment showed that the nsp2 of SCcd17 is 3195 nt in length and encodes 1064 amino acids (aa), with a discontinuous 131-aa deletion (111 + 1 + 19-aa) compared with the nsp2 of VR-2332, which is identical to that of NADC30-like strains (Figure 2). In addition, extensive variation sites were observed in SCcd17’s nsp2 compared with other representative strains (Table 3). SCcd17 shared amino acid identity of 66.3–66.4%, 64.9%, 67.3–67.4%, and 80.2–89.9%, respectively, with the nsp2 of JXA1-like, CH-1a-like, VR-2332-like, and NADC30-like PRRSV strains (Table 2).

**Figure 3:**
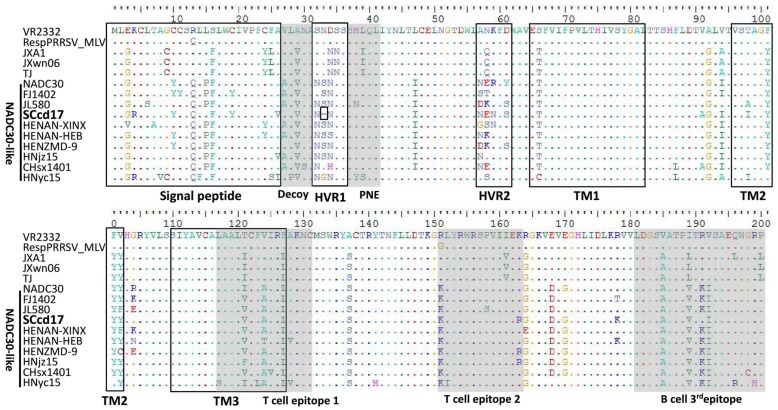
Multiple alignment of GP5 amino acid sequences of SCcd17 and fourteen reference strains. Black boxes indicate the regions of signal peptide, two hypervariable regions (HVR), and three transmembrane domains (TM). Gray areas indicate the amino acid residues in the decoy epitope, primary neutralizing epitope (PNE), two T cell epitopes and the 3^rd^ B cell epitope. The residues conserved were hidden.

ORF5 encodes a main envelope protein, GP5, which is considered the most heterogeneous structural protein. Sequences alignments of GP5 indicated that SCcd17 shares 83.0–83.6%, 84.2%, 81.2–81.8%, and 88.8–93.2% amino acid identity with JXA1-like, CH-1a-like, VR-2332-like, and NADC30-like strains, respectively (Table 2). Although ORF5 is the most variable region of PRRSV structural proteins, the deletion in this region is little recognized. Interestingly, amino acid alignment showed that the *ORF5* gene of SCcd17 encodes a 199-aa protein and has a novel 1-aa deletion in hypervariable region 1 (HVR1) at aa position 33, which is the first report of this deletion in GP5 (Figure 3). Additionally, comparison of the SCcd17 GP5 with the GP5 sequences of other strains revealed five putative *N*-glycosylation sites (identified by the sequence NXS/T, where X is any amino acid except proline) at aa positions 32, 34, 44, 51, and 58. The *N*-glycosylation site at aa position 58 is being reported for the first time in this study (Table 4). 

### 3.4. Recombination Analysis

**Figure 4:**
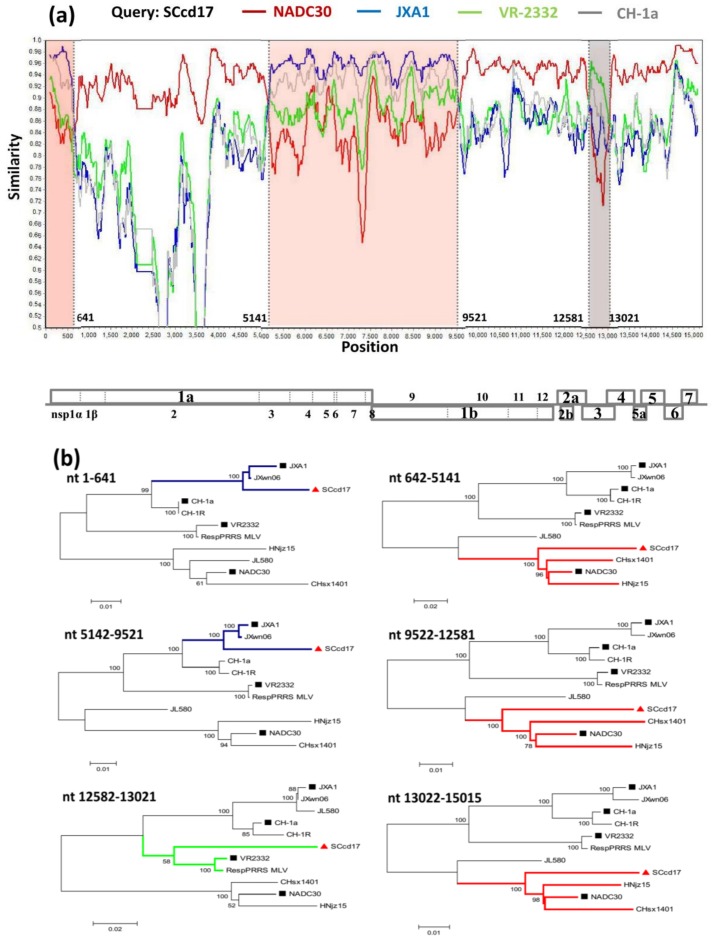
Recombination analysis of strain SCcd17. (**a**) Genome scale similarity comparisons of SCcd17 (query) with NADC30 (red), JXA1 (blue), VR-2332 (green), and CH-1a (gray). The six supposed recombination regions were showed with different colors and the recombination breakpoints were marked at the bottom with nucleotide sites and viral genome structure referenced to VR-2332; (**b**) phylogenetic trees based on every recombinant fragment within SCcd17 and ten reference PRRSV strains were shown below the similarity plot. The strain SCcd17 isolated was labeled with “red triangle”. The representative strains are labeled with “black squares”.

To identify possible recombinant events within SCcd17 strain, we performed recombinant detection using SimPlot and RDP software and the complete genome of SCcd17 as the query sequence. A similarity plot and phylogenetic analysis revealed that SCcd17 likely originated from multiple recombination events between the NADC30-like, JXA1-like, and VR-2332-like strains circulating in China (Figure 4a). From the similarity plot, five recombination breakpoints within the SCcd17 genome were identified, which were located in nsp1 (nt 641), nsp3 (nt 5141), nsp10 (nt 9521), ORF3 (nt 12,581), and ORF4 (nt 13,021). The five breakpoints separated the genome of SCcd17 into six regions. The two regions between the breakpoints (minor parental region, nt 1–641 and nt 5142–9521) are closely related to the JXA1-like strains, one region (minor parental region, nt 12,582–13,021) exhibited higher similarity with VR-2332-like strains, and the remaining three regions (major parental region, nt 642–5141, nt 9522–12,581, and nt 13,022–15,015) are closely related to the NADC30-like strains (Figure 4b). Collectively, the above results suggested that SCcd17 is a mosaic recombinant strain between a NADC30-like, a JXA1-like, and a VR-2332-like strain.

## 4. Discussion

HP-PRRSV has seriously affected the Chinese swine industry for decades since its outbreak in 2006. Previous study suggested that HP-PRRSV may have originated from the Chinese classic PRRSV (CH-1a-like), and gradually changed to the HP-PRRSV strain with the discontinuous 30-aa deletion in nsp2, resulting in a devastating virus that has caused immense economic losses in China [33]. However, later study demonstrated that this unique deletion is not related to the virulence of PRRSV [34]. Although commercial MLV vaccines (e.g., JXA1-P80, TJMF-92, R98, and HuN4-F112) against HP-PRRSV are widely used, current control strategies have failed to provide sustainable disease control due to the extensive genetic variation and diversity of PRRSV. Possibly, the extensive use of the MLV vaccines might have increased the immune selective pressure in pig herds to accelerate the variation and evolution of PRRSV [18,25,35,36]. In 2013, the NADC30-like PRRSV strains emerged in China, increasing the complexity of PRRS in the field. In the following one to two years, many recombinant strains between NADC30-like and Chinese HP-PRRSVs/VR-2332 were reported in China, including HENAN-HEB, JL580, HNjz15, HNhx, TJnh1501, FJ1402, Chsx1401, and HNyc15 [19,20,21,26,28,30,37,38]. Our previous study showed three sub-genotypes (JXA1-like, VR-2332-like, and NADC30-like) of genotype 2 PRRSV were the predominant viruses in southwestern China during 2012–2016 [32]. In the present study, a novel PRRSV variant, designated SCcd17, was isolated in 2017 in southwestern China, and its genomic characteristics were analyzed. The results revealed that the SCcd17 isolate is a multiple recombinant strain among NADC30-like, JXA1-like, and VR-2332-like strains recently prevalent in the region. The SCcd17 strain displayed a different recombination pattern compared with the reported NADC30-like strains isolated from 2013 to 2016 in China (Table 5), indicating the emergence of new PRRSV recombination variant in China. The genomic analysis of SCcd17 revealed that recombination events of PRRSV in China were extremely complex.

Phylogenetic analysis based on the complete nucleotide sequences indicated that Chinese genotype 2 PRRSVs were mainly clustered into four sub-genotypes: JXA1-like, CH-1a-like, VR-2332-like, and NADC30-like sub-genotypes. Furthermore, NADC30-like strains in China were classified into two different branches (Clusters 1 and 2), indicating the presence of NADC30-like PRRSVs with genetic diversity in China. SCcd17 is most closely related to cluster 2 of NADC30-like sub-genotype strains, however, shared low nucleotide identity (88.1–90.2%) with NADC30-like strains due to the uptake of nucleotide sequences of JXA1-like strain (nt 1–641, 5141–9521) and VR-2332-like strain (nt 12,581–13,021).

*Nsp2* and *ORF5* gene are frequently used as molecular markers to monitor the molecular epidemiology and evolution of PRRSV strains [23,24,25,39,40,41,42]. NADC30-like PRRSV has a unique discontinuous 131-aa deletion in nsp2-coding region relative to the sequence of VR-2332, which differs from the molecular marker of JXA1-like HP-PRRSVs (30-aa deletion) [14,43]. In this study, the SCcd17 strain lacks that discontinuous 131-aa sequence in nsp2, suggesting that SCcd17 strain is an NADC30-like strain and the 131-aa deletion could be used as an epidemiological molecular marker for the NADC30-like PRRSV circulating in China. Nsp2 is crucial for viral replication and the modulation of host immunity, and nsp2 contains many immunogenic epitopes [44,45]. Our data showed that there are a number of amino acid substitutions in SCcd17’s nsp2. Whether these mutations are associated with virulence requires further study.

ORF5 is the most variable PRRSV structural protein. Notably, there is a unique amino acid deletion at position aa 33 of GP5, a change first described in SCcd17 (Figure 3). The potential *N*-glycosylation sites at aa positions 44 and 51 are mostly conserved within the four sub-genotype PRRSV strains. Most NADC30-like sub-genotype PRRSV isolates possess three potential *N*-glycosylation sites, however, SCcd17 has five *N*-glycosylation sites, with one novel *N*-glycosylation site (aa 58) identified (Table 4). Previous study has shown that *N*-glycosylation may increase the number of the N-linked glycans, hindering antibody evasion, but it does not affect the ability of the virus to bind to cellular receptors in the host [46,47]. Therefore, the increased number of *N*-glycosylation sites in GP5 suggests that the SCcd17 isolate may possess the capability to escape the neutralization antibodies induced by MLV vaccines. In addition, all the NADC30-like strains in China had two substitutions of R^13^→Q^13^ and R^151^→K^151^ (Figure 4), changes that may be associated with the virulence of PRRSV [48].

Recombination and mutation are pervasive among PRRSV isolates and are the major mechanisms contributing to the emergence and evolution of new PRRSV variants [16,18,48,49]. The Chinese HP-PRRSV started from CH-1a to NB/04 with one aa deletion, and eventually acquired discontinuous 30-aa (1 + 29-aa) deletion in nsp2 [33]. The recent NADC30-like PRRSV strains in China were reported to show significant genetic recombination and mutation [19,20,21], and the new NADC30-like recombinant PRRSV isolate SCcd17 shows evidence of recombination between three sub-genotypes strains of type 2 PRRSV, suggesting PRRSV strains in China have undergone rapid evolution in recent years. Considering the isolation years and genetic characteristics of these new PRRSV strains which have emerged in China in recent years, the SCcd17 isolate may have had a complicated evolutionary progress, starting from the recombination of a NADC30-like recombinant strain with Chinese HP-PRRSV, and then evolution to a new multiple recombinant strain with a VR-2332-like virus. A previous study demonstrated that nsp9 and nsp10 together contribute to the fatal virulence of HP-PRRSV [50]. In this study, NADC30-like SCcd17 contains some sequences of the HP-PRRSV strain, involving a complete *nsp9* and partial *nsp10* gene. The pathogenesis of this novel recombinant isolate requires further investigation. Nevertheless, the genomic data of SCcd17 suggests that the PRRSVs in China have continuously evolved and new vaccine strategies are necessary for more efficient control of the virus.

## 5. Conclusions

In summary, we isolated a novel NADC30-like PRRSV strain, SCcd17, with unique genetic variation in GP5 and nsp2. The genomic sequence analysis revealed evidence of multiple recombination events between NADC30-like, JXA1-like, and VR-2332-like strains that are prevalent in China. Coupled with the tendency for recombination and mutation, our results suggest the likelihood of future outbreaks of new PRRSV variants and highlight the need for continuous viral surveillance.

## Figures and Tables

**Table 1:** Information on the reference porcine reproductive and respiratory syndrome viruses (PRRSVs) downloaded from GenBank.

Reference Strains	Country/Year	Accession No.	Reference Strains	Country/Year	Accession No.
VR-2332 ^abc^	USA/1995	AY150564	WUH4 ^a^	CHN/2012	JQ326271
CH-1a ^abc^	CHN/1996	AY032626	HENAN-XINX ^a^	CHN/2013	KF611905
BJ-4 ^a^	CHN/2000	AF331831	JL580 ^ab^	CHN/2013	KR706343
RespPRRS MLV ^ab^	USA/2005	AF066183	XW015 ^a^	USA/2013	KF724409
JXA1 ^abc^	CHN/2006	EF112445	FJ1402 ^a^	CHN/2014	KX169191
TJ ^ab^	CHN/2006	EU860248	CHsx1401 ^ab^	CHN/2014	KP861625
MN184A ^a^	USA/2006	DQ176019	HNjz15 ^ab^	CHN/2015	KT945017
jiangxi-3 ^a^	CHN/2007	EU200961	FJZ03 ^a^	CHN/2015	KP860909
07BJ ^a^	CHN/2007	FJ393459	HNyc15 ^a^	CHN/2015	KT945018
CH-1R ^a^	CHN/2008	EU807840	FJY04 ^a^	CHN/2015	KP860910
NADC30 ^abc^	USA/2008	JN654459	WUH5 ^a^	CHN/2015	KU523366
JXwn06 ^ab^	CHN/2008	EF641008	HENZMD-9 ^a^	CHN/2015	KU950374
JXA1 P80 ^a^	CHN/2008	FJ548853	HNhx ^a^	CHN/2016	KX766379
HENAN-HEB ^ab^	CHN/2012	KJ143621			

^a^ PRRSV sequences used in phylogenetic analysis; ^b^ PRRSV sequences used in sequence identity analysis; ^c^ PRRSV sequences used in recombination analysis.

**Table 2:** Nucleotide and amino acid identities of different regions of SCcd17 compared with other PRRSV reference strains.

	JXA1	JXwn06	TJ	VR-2332	RespPRRSV MLV	CH-1a	NADC30	JL580	CHsx1401	HENAN-HEB	HNjz15	LV
	JXA1-Like			VR-2332-Like		CH-1a-Like	NADC30-Like					Type 1
	Pairwise % Identity to SCcd17 (nt/aa)											
Complete genome	85.2	85.3	92.7	84.0	83.9	84.9	**90.2**	88.3	88.1	87.6	88.3	43.4
5′UTR	**97.8**	**97.8**	94.9	92.7	92.7	96.7	92.2	91.5	90.3	92.2	90.9	49.2
ORF1a	82.1	82.4	82.1	79.9	79.8	81.0	**88.5**	86.6	86.1	84.1	86.1	32.5
ORF1b	94.3	**94.4**	94.1	89.1	89.0	92.3	85.6	84.5	84.7	84.7	83.6	50.0
nsp1α	97.3/**97.7**	**98.1/97.7**	**98.1/97.7**	89.7/96.0	89.7/96.0	94.8/96.0	86.2/94.8	86.5/96.0	83.5/94.8	85.3/94.3	85.5/94.8	49.4/43.3
nsp1β	88.4/77.9	88.4/77.9	88.4/77.9	89.1/77.9	88.9/77.3	87.5/74.8	**94.4/85.7**	93.3/83.9	93.7/84.5	92.6/83.3	92.2/**85.7**	31.9/25.6
nsp2	67.1/66.3	67.0/66.4	66.5/66.4	71.7/67.4	71.6/67.3	68.1/64.9	**92.3/89.9**	82.7/80.2	89.0/85.5	85.0/82.0	89.6/87.4	23.6/20.9
nsp3	89.1/92.3	88.9/92.8	88.8/92.3	87.8/92.3	87.7/92.3	88.1/91.8	**90.4/94.2**	93.4/**94.2**	90.0/93.7	90.8/93.7	90.4/**94.2**	32.8/22.7
nsp4	97.8/**98.0**	**97.9/98.0**	**97.9/98.0**	92.4/92.9	92.4/92.9	95.8/94.4	88.1/91.8	97.0/97.0	87.6/92.3	86.9/90.2	87.8/91.3	44.8/54.9
nsp5	96.4/**95.8**	**97.0/95.8**	96.8/**95.8**	89.9/88.1	89.9/88.1	95.0/92.6	91.5/92.6	93.3/92.0	89.1/88.1	89.5/90.7	88.0/86.1	50.9/38.9
nsp6	**97.9/100**	**97.9/100**	95.6/**100**	93.3/93.1	93.3/93.1	95.5/**100**	95.6/93.1	**97.9/100**	82.7/85.7	90.6/93.1	92.1/93.1	58.1/69.0
nsp7α	96.3/87.3	**96.8**/87.3	**96.8**/87.3	87.4/84.4	87.4/84.4	94.6/95.9	80.1/90.8	92.3/**97.3**	79.7/89.3	77.7/88.6	79.2/90.8	29.6/42.7
nsp7β	95.3/96.3	**96.0/97.2**	95.6/96.3	81.7/76.3	81.7/76.3	90.9/91.4	73.2/67.8	73.9/72.7	71.9/65.2	72.1/66.5	68.8/63.9	22.8/20.9
nsp8	96.2/**97.7**	**97.0/97.7**	**97.0/97.7**	92.8/**97.7**	92.8/**97.7**	**97.0/97.7**	86.9/**97.7**	85.9/**97.7**	86.8/95.3	82.0/92.9	83.0/92.9	48.8/65.0
nsp9	96.0/98.7	**96.1/99.0**	95.8/**99.0**	89.4/97.4	89.4/97.4	93.6/98.1	84.3/97.2	83.2/96.3	83.9/96.3	83.5/96.3	82.5/96.6	54.0/69.8
nsp10	82.9/94.4	83.0/95.1	82.8/94.6	85.8/95.6	85.5/95.6	84.1/94.4	**94.6**/95.1	93.3/97.9	92.2/**98.2**	93.5/97.5	92.6/97.7	46.4/57.8
nsp11	88.2/94.4	88.6/94.9	88.2/94.4	86.9/92.5	87.6/94.0	91.1/94.4	94.5/94.9	92.2/94.4	88.4/93.5	**94.6/95.4**	92.3/94.4	53.5/74.5
nsp12	86.3/95.3	86.3/95.3	86.3/95.3	85.0/93.2	85.3/93.2	87.2/93.9	**95.7**/97.3	94.3/98.7	95.0/96.7	95.2/**98.0**	95.2/97.3	24.3/15.5
ORF2a	84.3/85.2	84.6/85.2	84.6/85.2	88.3/89.7	88.0/89.2	86.1/87.9	**92.8**/92.3	91.8/91.4	90.9/91.0	91.6/90.1	92.3/**93.1**	52.4/62.0
ORF2b	86.9/85.3	86.9/85.3	86.985.3	90.7/86.8	90.2/86.8	86.5/83.7	93.9/91.4	94.9/94.4	92.9/91.4	93.4/92.9	**96.3/94.4**	65.2/67.1
ORF3	83.2/80.0	83.0/80.5	83.2/80.5	88.2/83.8	88.6/84.7	85.2/81.9	84.5/84.3	82.7/80.0	84.5/**85.2**	93.7/83.8	83.2/84.3	52.7/47.1
ORF4	80.5/82.9	81.9/84.9	81.7/85.5	82.5/82.2	82.5/82.2	82.7/84.9	**94.3/92.4**	84.8/84.9	92.9/91.8	92.7/90.0	92.2/90.6	58.5/61.4
ORF5	88.8/83.0	89.0/83.6	89.0/83.6	89.1/81.2	89.2/81.8	90.2/84.2	**96.4**/92.7	94.9/88.8	95.4/91.6	95.3/91.6	95.7/**93.2**	46.2/42.2
ORF6	87.0/93.5	86.8/93.5	86.8/93.5	88.1/94.1	88.4/94.1	85.9/92.2	**96.9/98.3**	95.6/97.1	95.4/98.3	96.1/98.3	95.6/98.3	60.4/78.8
ORF7	87.7/87.9	85.6/83.2	87.7/87.9	90.4/91.5	90.4/91.5	89.0/89.7	**97.2/96.7**	95.5/94.1	94.3/92.4	94.9/94.1	95.8/95.9	55.0/51.7
3′UTR	89.2	82.3	89.2	93.0	93.0	88.5	**97.3**	**97.3**	96.6	96.6	96.6	56.0

The highest nucleotide and amino acid identities of different regions are indicated in bold typeface.

**Table 3:** Analysis of amino acid mutations in nonstructural protein 2 (nsp2).

Strains	Amino Acid Sites																					
	29	35	76	120	152	193	267	300	314	470	485	534	553	608	610	636	785	846	886	924	958	1093
VR-2332	A	A	P	E	L	S	E	Q	V	L	S	P	Q	E	S	K	Q	S	F	G	D	R
RespPRRSMLV	A	A	P	E	L	S	E	Q	V	M	S	P	Q	E	S	K	Q	S	F	G	D	R
CH-1a	A	A	P	E	L	S	E	Q	V	M	G	P	Q	G	S	K	Q	S	F	G	D	R
JXA1	A	A	P	E	L	S	E	Q	V	M	G	DL	DL	E	S	K	Q	S	F	G	D	R
JXwn06	A	A	P	E	L	S	E	Q	V	M	G	DL	DL	E	S	K	Q	S	F	G	D	R
TJ	A	A	P	E	L	S	E	Q	V	L	G	DL	DL	E	S	K	Q	S	F	G	D	R
NADC30	A	A	P	E	L	F	E	Q	V	L	G	L	Q	E	S	K	Q	S	F	G	D	R
CHsx1401	A	A	P	E	L	S	E	Q	V	L	G	L	Q	E	S	K	Q	S	F	G	D	R
JL580	A	A	P	E	L	F	E	Q	V	L	G	L	Q	E	S	K	Q	S	F	G	D	R
HENAN-HEB	A	A	P	E	L	S	E	Q	V	L	G	L	Q	E	S	K	P	S	F	G	D	R
HNjz15	A	A	P	E	L	S	E	Q	V	L	G	L	Q	E	S	K	Q	N	F	G	D	R
SCcd17	T	V	S	R	S	I	D	R	A	S	A	S	R	K	A	R	R	I	L	S	E	K

DL: amino acid deletion.

**Table 4:** Potential *N*-glycosylation sites in GP5.

Strains	Potential *N*-glycosylation Sites							
	30	32	33	34	35	44	51	58
VR-2332	+	-	+	-	-	+	+	-
RespPRRS MLV	+	-	+	-	-	+	+	-
CH-1a	-	-	-	+	-	+	+	-
JXA1	+	-	-	+	+	+	+	-
JXwn06	+	-	-	+	+	+	+	-
TJ	+	-	-	+	+	+	+	-
NADC30	-	-	-	+	-	+	+	-
CHsx1401	-	-	-	+	-	+	+	-
JL580	-	-	-	+	-	+	+	-
HENAN-HEB	-	+	-	-	-	+	+	-
HNzj15	-	-	-	+	-	+	+	-
SCcd17	-	+	-	+	-	+	+	+

“+” indicates the presence and “-” indicates the absence of *N*-glycosylation sites.

**Table 5:** Recombination analysis of NADC30-like PRRSVs isolated in China during 2013–2017.

Strains	Isolation Date	Recombination with	Recombination Regions	Accession No.	References
HENAN-HEB	2013	JXA1	nsp2	KJ143621	[29]
HENAN-XINX	2013	VR-2332	nsp2–5	KF611905	[29]
JL580	2014	09NEN1 (JXA1-like)	nsp2, nsp3, nsp7, ORF2a, ORF4	KR706343	[19]
Chsx1401	2014	VR-2332	nsp11	KP861625	[29]
FJ1402	2014	GD (JXA1-like)	nsp2–3, nsp12, ORF3	KX169191	[27]
HENZMD-9	2015	JXA1	5′-UTR-nsp2, nsp4–9	KU950374	[30]
HNyc15	2015	VR-2332/CH-1a	ORF2–4	KT945018	[26]
15HEN1	2015	JXA1-R ^a^	nsp3–9	KX815413	[38]
15LN3	2015	JXA1-R ^a^	nsp3–9	KX815425	[38]
15SC3	2015	JXA1-R ^a^	5′-UTR-nsp2, nsp7–nsp9	KX815428	[38]
TJnh1501	2015	TJbd14-1(JXA1-like)	nsp2	KX510269	[28]
SCnj16	2016	JXA1	5′-UTR, nsp1–2, nsp3–9	MF196906	[32]
HNhx	2016	JXA1	nsp4–9	KX766379	[30]
SCcd17	2017	JXA1+VR-2332	JXA1(5′-UTR-nsp1α, nsp3–9) VR-2332 (ORF3–4)	MG914067	This study

^a^ Modified live-attenuated vaccine.
